# Epidemiological characteristics and outcomes of the 2023–2024 cholera outbreak in South Yemen: a retrospective descriptive study from MSF-supported centres

**DOI:** 10.1186/s12889-026-27771-w

**Published:** 2026-05-14

**Authors:** Masar Sallam, Noor Othman Abdulgany, Abdulhakeem Moh Farhan, Moath Shomasi, Ahmed Abdulhaq, Umar Zaman Khan, Darwin Dela Cruz-Diaz, Hilde De Clerck, Sylvia Lim, Krystel Moussally, Pilar Garcia-Vello

**Affiliations:** 1 Yemen Mission, Médecins Sans Frontières OCB, Aden, Yemen; 2 Sadaqa Hospital cholera treatment centre, Ministry of Health (MoH), Aden, Yemen; 3 Mocha Mother and Child Hospital cholera treatment centre, Médecins Sans Frontières OCB, Mocha, Yemen; 4 Mafraq Primary Health Care Center cholera treatment unit, Médecins Sans Frontières OCB, Mafraq, Yemen; 5 Ataq cholera treatment centre, Médecins Sans Frontières OCB, Ataq, Yemen; 6 Medical Department, Médecins Sans Frontières OCB, Brussels, Belgium; 7 Luxembourg Operational Research and Epidemiology Support Unit (LuxOR), Médecins Sans Frontières OCB, Luxembourg, Luxembourg; 8 Middle East Medical Unit (MEMU), Médecins Sans Frontières OCB, Beirut, Lebanon

**Keywords:** Cholera, Outbreak, Malnutrition, Middle-East, Conflict, Yemen

## Abstract

**Background:**

The cholera burden in Yemen is the highest in the world, with the country facing recurrent outbreaks. It is worsened by the ongoing conflict, limited access to clean water, and the deteriorated sanitation and health systems. In November 2023, a cholera outbreak started in Shabwah governorate and seemed to be controlled in January 2024. However, in March 2024, cholera spread westward, causing 40,000 suspected cases in South Yemen.

**Methods:**

Routinely collected data were used to provide descriptive statistics of the 2023–2024 cholera outbreak of patients managed in the Médecins Sans Frontières (MSF)–supported cholera treatment centers (CTCs) and units (CTUs) of Aden, Ataq, Mafraq and Mocha.

**Results:**

There were 10,252 suspected cholera cases admitted to MSF-supported facilities. Of these, 52.5% were male, and 41.4% were aged 15–44 years and 24.1% 0 to 4 years. Most cases were managed in Aden (56.2%) and Mocha (24.8%). Of these, 43.1% were classified as non-dehydrated (plan A), 41.9% had moderate dehydration (plan B), and 15.0% had severe dehydration (plan C). The overall case fatality rate in MSF-supported facilities was 0.2% compared to 0.52% across South Yemen. Aden’s Sadaqa CTC treated 80.3% of all Plan C cases, reflecting its role as a referral hub.

**Conclusion:**

The 2023–2024 cholera outbreak highlighted persistent challenges in South Yemen, such as conflict, inconsistent laboratory testing and surveillance, and limited access to healthcare, despite a low case fatality rate. Outbreak preparedness must shift from reactive to anticipatory, including strategic storage of medicines, vaccines and diagnostics and clear guidelines on how to use them.

## Background

The cholera burden in Yemen is the highest in the world, due to recurrent outbreaks [1]. It is worsened by the ongoing conflict, the limited access to clean water, and the deteriorated sanitation and health systems [2]. Since 2014, the armed conflict has led to population displacement, destruction of water and sanitation infrastructure, and constraints to access to healthcare. The current global resurgence of cholera, declared a grade 3 emergency by the World Health Organization (WHO) in 2022, has affected more than 30 countries, including Yemen [3]. Within this wider context, Yemen’s latest cholera outbreak began in November 2023 in Shabwah governorate, with cases gradually declining by January 2024. However, in March 2024, a new surge in cholera cases was observed spreading westward. From January 1 to December 29, 2024, Yemen reported a total of 260,552 cholera cases and 879 associated deaths, with an estimated attack rate (AR) of 0.78%, and a case fatality rate (CFR) of 0.34% [4, 5]. In the South, Yemen’s electronic Disease Early Warning System (eDEWS) recorded about 40,000 suspected cases from March to October 2024, with an AR of 0.32% and a CFR of 0.52% [6]. The eDEWS is a national surveillance system to which public and selected private health facilities report notifiable diseases.

Cholera is not new to Yemen, from October 2016 to December 2020, more than two million suspected cases and more than 3,500 deaths were reported [7–10]. While rapid access to appropriate hydration is the mainstay of therapy, severe cases need antibiotics. However, most *Vibrio cholerae* isolates belonged to serogroup O1 and were resistant to third-generation cephalosporins and macrolides (including azithromycin) [11], which limits treatment options.

Children in Yemen are especially at risk. Not only is young age a well-known risk factor [12–14], but malnutrition significantly increases the risk of contracting diarrheal illness and having more severe, prolonged and fatal disease [12–16]. As it is estimated that 2.7 million children in Yemen are acutely malnourished and 49% of children under 5 years old suffer from stunting or chronic malnutrition, the risks are compounded [17].

In response to the 2023–2024 outbreak, 19 cholera treatment centres (CTC)/cholera treatment units (CTU) and 198 oral rehydration points (ORP) were established in South Yemen. CTCs provide full rehydration and monitoring capacity for inpatient care of severe cases; CTUs offer stabilisation for less severe cases; and ORPs are community-based structures for oral rehydration of mild cases. In addition, in November 2024, 3.8 million individuals aged one year and above received the oral cholera vaccine (OCV) in urban and rural areas of Aden, Abyan, Al-Dhale, Lahj, Marib, and Taiz [18], which adds to the previous campaigns of 2018 (reaching 270,000 people) [19]; 2019 (400,000 people) [20], 2020–2021 and 2022–2023 (2.1 million people) [21, 22].

Médecins Sans Frontières (MSF), present in Yemen since 1986, has responded to the outbreak in Aden, Shabwa and Taiz governorates. This manuscript describes the cholera outbreak among patients admitted to the MSF CTCs/CTUs in Aden (Aden governorate), Ataq (Shabwa governorate), Mafraq and Mocha (Taiz governorate) in 2023–2024, with a particular focus on operational response, case management, and challenges encountered in a conflict-affected setting.

## Methods

### Study design and population

We conducted a retrospective cohort analysis using routinely collected clinical and epidemiological data from the MSF CTCs and CTU in Aden, Ataq, Mafraq and Mocha, between April – October 2024. All patients who were seen and/or admitted to any of the MSF study centres with suspected or confirmed cholera were included in the study. Patients arrived either through self-referral or after referral by a nearby hospital or ORP.

### Study setting

**Fig. 1:**
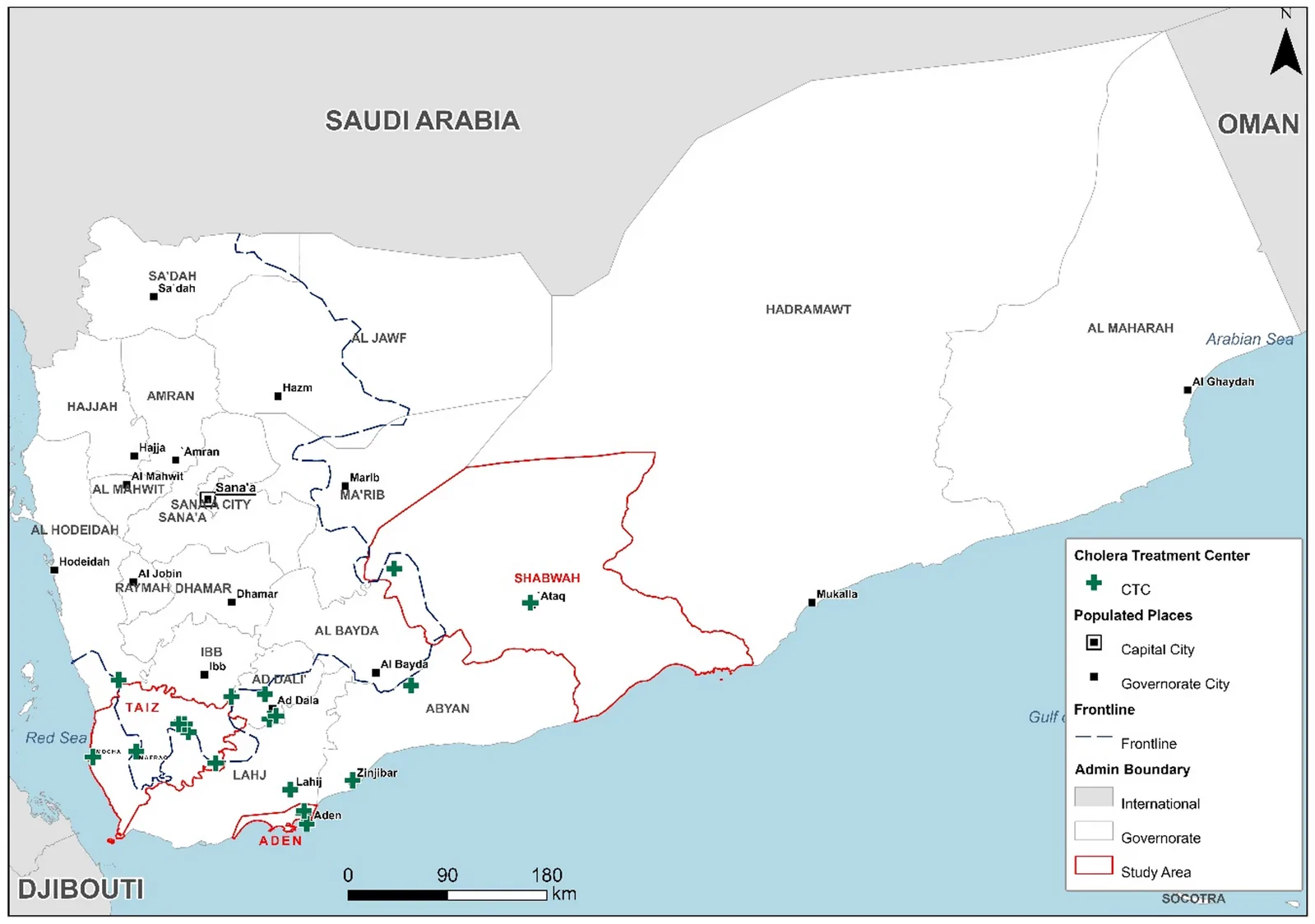
Map of cholera treatment centres in South Yemen, 2024

Since the onset of the protracted conflict in September 2014, Yemen has remained politically divided. This study describes the cholera response in southern Yemen (Fig. 1). The characteristics of the MSF-supported CTCs and CTU in Aden, Ataq, Mafraq and Mocha are summarised in Table 1.

**Table 1:** Key characteristics of MSF-supported cholera treatment sites in South Yemen, 2024

Site	Facility type	Bed capacity	Catchment characteristics	Referral role	Diagnostic capacity
Aden (Sadaqa)	CTC	86 beds	Urban, high population density	Major referral hub (receives severe cases)	Limited, inconsistent RDT/culture
Ataq	CTC (MoH-supported)	20 beds	Semi-urban, dispersed population	Localised care, limited referrals	High RDT/culture capacity
Mocha	CTC	20 beds	Rural/coastal, conflict-affected	Receives referrals (including from Mafraq)	Limited, inconsistent RDT
Mafraq	CTU (PHCC-linked)	4 beds + 5 chairs	Rural, low-access setting	Stabilisation + referral to Mocha	No testing capacity

*CTC* cholera treatment center, *CTU* cholera treatment unit, *RDT* rapid diagnostic test

In Aden governorate (1,050,000 inhabitants), MSF supported an 86-bed CTC at Sadaqa Hospital in collaboration with the Ministry of Health (MoH) and the International Organization for Migration (IOM). This CTC was the primary cholera management facility in Aden governorate. Sadaqa CTC was located in Dar Sad district (37 km^2^; 153,000 inhabitants), a densely populated urban and residential area on the outskirts of central Aden with an AR of 1.11% and CFR of 0.62% in 2024 [6]. The district is characterised by rapid urban expansion, limited infrastructure, and vulnerabilities related to displacement, as it hosts many internally displaced persons (IDPs) due to ongoing conflict. Additionally, the governorate had another CTC/CTU in the Al Muralla district and 29 ORPs (Fig. 1).

In Shabwa governorate (676,408 inhabitants), MSF has been supporting Ataq’s mother and child hospital’s emergency room (ER), the in-patient and out-patient departments (IPD and OPD), intensive care unit (ICU), and the high-dependency and isolation wards since 2022 (Fig. 1). Near the hospital, MSF supported the only cholera treatment facility in the district, a 20-bed MoH CTC. Data from one MoH CTC (supported by MSF) and two ORPs showed an AR of 4.39% and a CFR of 0% in 2024 [6]. Ataq district (1,300 km^2^; 56,000 inhabitants) is undergoing gradual urban expansion and has experienced significant population movements due to the conflict.

In Taiz governorate (3,100,000 inhabitants), MSF works in two projects in Mafraq and Mocha (Fig. 1).

In Mocha, Al Makha district (1,617 km^2^; 80,000 inhabitants), MSF has supported a paediatric IPD, isolation, and a comprehensive emergency obstetric and newborn care (CEmONC) hospital since 2022. Nearby, MSF supported the only 20-bed CTC in Al Makha district. Data from one MoH CTC (supported by MSF) and four ORPs showed an AR of 3.26% and CFR of 0.34 in 2024 [6]. Al Makha is a coastal district with a relatively sparse population spread across rural and semi-urban settlements. During 2024, the governorate experienced the reactivation of the nearby frontline, the movement of IDPs and the influx of arrivals from Ethiopia.

In Mafraq, Mawza district (665 km^2^; 120,000 inhabitants), MSF has been supporting an MoH Primary Health Care Center (PHCC) since 2022. Nearby, MSF assisted the only 4-bed and 5-chair CTU. Mawza district is a rural area located in the western part of Taiz governorate with an AR of 4.24%, CFR of 3.3%, for Mawza in 2024 [6]. The Mawza district was equipped with two additional ORPs but lacked a CTC; consequently, patients presenting with more severe conditions were referred to Mocha district for advanced treatment when required.

Following the detection of the first cholera case in September 2023 in Ataq, MSF collaborated with the MoH to respond to the outbreak by providing logistical and medical support. The collaboration continued with the establishment of CTCs in Ataq, Aden, and Mocha, as well as a CTU in Mafraq.

MSF’s intervention included case management, health promotion, water and sanitation, and infection prevention and control. Health promotion was integrated into the medical response. MSF delivered health education to patient relatives and hospital staff (including ER, OPD, and ICU teams), focusing on cholera case definitions, signs and symptoms, transmission routes, treatment, prevention, and referral pathways. MSF prioritised outreach in Dar Sa’d district—consistently the main source of cases in Aden—by conducting door-to-door awareness campaigns in collaboration with community leaders. Additionally, MSF launched digital health promotion, targeting residents in all governorates with standardised messaging.

In addition, UNICEF shared educational materials on cholera prevention in April and May 2024 [23] and supported a mass oral cholera vaccination campaign in November 2024, covering Aden and Taiz governorates [18].

### Case and disease severity definitions

Suspected case was defined, in line with MoH definitions [24], as a person aged two years or older: with acute watery diarrhoea and severe dehydration; or who died from acute watery diarrhoea with no other known cause of death. In addition, all children under two presenting at MSF-supported CTCs/CTUs with acute watery diarrhoea were managed as suspected cholera and included in the study database. Confirmed case was defined as any person infected with *Vibrio cholerae* O1 or O139, as confirmed by culture (including seroagglutination) or Polymerase Chain Reaction. Rapid diagnostic test (RDT) alone is not considered confirmation [24]. To monitor the outbreak, Abbott Bioline™ Cholera Ag O1/O139 RDT and stool cultures through the thiosulfate citrate bile salts sucrose agar (TCBS) method were performed on a random subset of suspected cases by the MoH. The criteria or procedures used to select this random subset were not formally documented, moreover, testing capacity varied by location and over time.

Cases were treated as per MSF clinical guidelines [25]. Severity definition and treatment are as follows:

1. *Severe dehydration* was defined as at least one danger sign or at least two of the following: very sunken eyes, skin pinch very slowly to disappear (> 2 s.), or the patient drinks very little. Severe dehydration patients were treated with plan C, which is a volume of Ringer lactate corresponding to 10% of the patient’s body weight.
2. *Moderate dehydration* was defined as no danger signs and at least two of the following: eyes lightly sunken, skin pinch that disappears slowly (< 2 s.), or the patient was very thirsty and drinks avidly. Moderate dehydration was treated with plan B, which is a volume of oral rehydration solution (ORS) corresponding to 5–9% of their body weight.
3. *Non-dehydration* was defined by the lack of signs of severe or moderate dehydration. These patients were treated with plan A at the CTC/CTU/ORP or were maintained with ORP at home, when possible. For children under 5 years old, admission for observation was recommended. Complementary therapy: empirical doxycycline 4 mg/kg oral for children over 1 year of age and a single oral dose of 300 mg for adults with severe or moderate dehydration, administered within the first 4 h of treatment, or as soon as possible once the patient is hemodynamically stable and can tolerate oral therapy; Zinc supplementation is recommended for all children under 5 years of age presenting with diarrhoea, regardless of dehydration status. Oral zinc sulfate is recommended in a 10-day course, 10 mg once daily for children under 6 months, 20 mg once daily for children between 6 months and 5 years.

### Data sources

Epidemiological and clinical data were collected using a standardised Excel linelist. Data encoders updated the linelist weekly using information from on-site paper-based registries. The linelist was implemented for routine use in all MSF-supported locations throughout the outbreak response period. Collected variables included socio-demographic data, cholera vaccination status (number of doses and date), clinical data including admission information such as date of symptom onset, malnutrition status for children assessed using routine MSF protocols [26, 27], source of referral, and laboratory-related data including RDT and culture results, bacterial strain and resistance patterns. In addition, data on treatment (rehydration plan, antibiotics or supplements) and patient outcomes (discharge, death, left against medical advice, referral) were also included.

### Data analysis

Descriptive statistics were used to summarise patient characteristics, including all suspected and confirmed cases reported in the linelist. For categorical variables, frequencies and proportions were reported. For continuous variables, we used either medians and ranges. A temporal analysis was conducted using epidemic curves to identify trends over time. Attack rates (AR) and case fatality rates (CFR) were calculated using population denominators and mortality data obtained from routine surveillance sources, as described in the study setting. No inferential statistical testing was performed. Variables with a ≥ 5% of missing values were not reported; no imputation or other treatment of missing data was performed. Analysis was conducted using R-4.3.0. Maps were generated using data from MSF and OpenStreetMap through the GeoMSF platform, supported by the MSF Middle East GIS Unit.

### Ethics

This research fulfilled the exemption criteria set by the Médecins Sans Frontières Ethics Review Board for *a posteriori* analyses of routinely collected clinical data. The requirement for informed consent to participate was waived by the MSF Ethics Review Board, as the study involved secondary analysis of anonymised routine programmatic data. In addition, the study received approval for publication from the Minister of Health of Yemen.

## Results

### General outbreak description

**Fig. 2:**
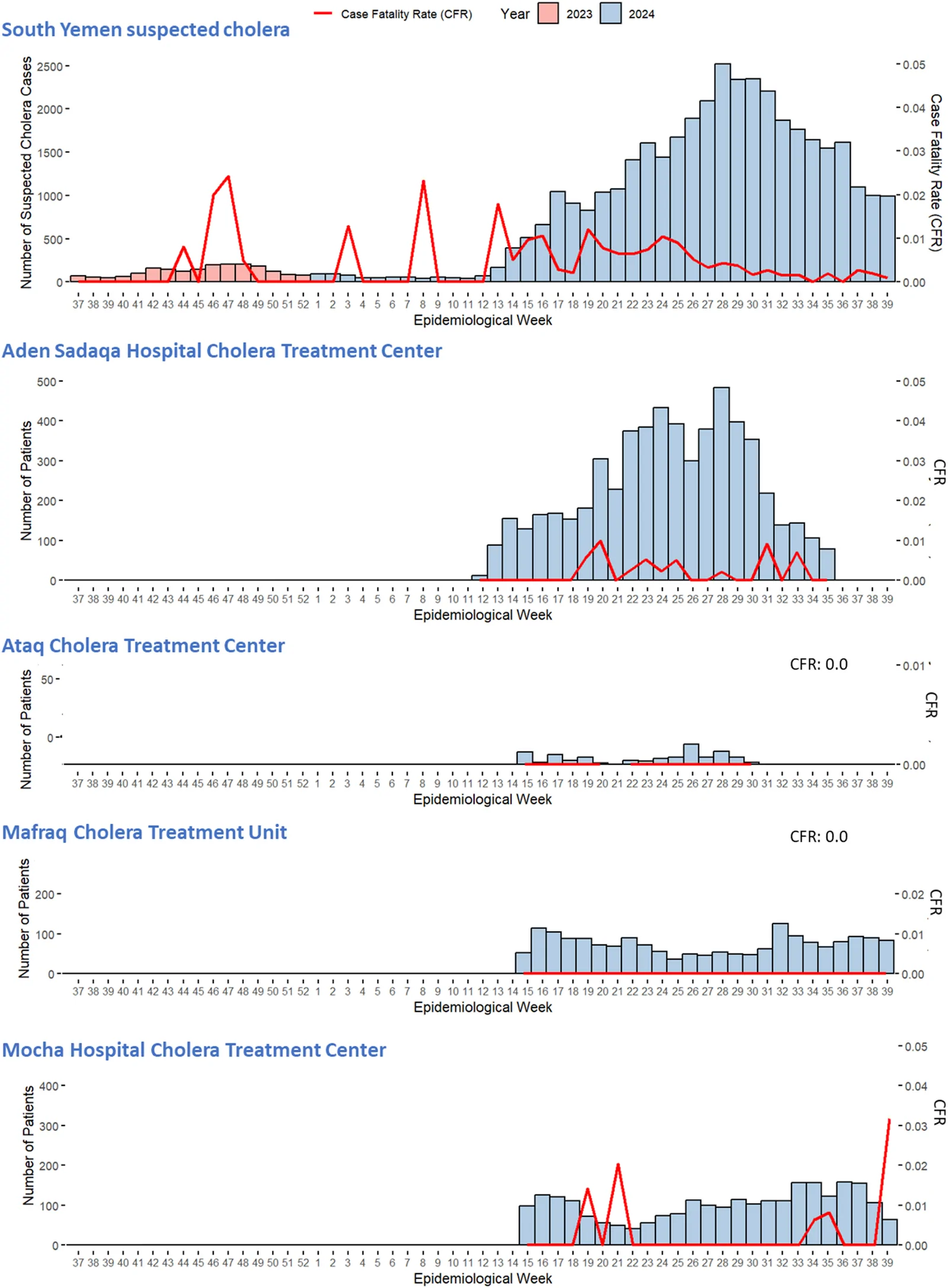
Suspected cholera cases and case-fatality rate in South Yemen as reported by Electronic Disease Early Warning System in Yemen (eDEWS), and MSF-supported facilities in Aden, Ataq, Mafraq, and Mocha during the 2023–2024 outbreak

Between April and October 2024, 10,252 suspected cases were treated at MSF-supported facilities in Aden, Ataq, Mafraq, and Mocha. This represents 25.6% of the total suspected cases of South Yemen. Sadaqa Hospital CTC in Aden managed almost half of the cases (56.2%), followed by Mocha Mother and Child Hospital CTC (24.8%) (Fig. 2; Table 2). Of the suspected cases seen in the study centres 5,375 (52.5%) were male, and most of them were aged 15 to 44 years old (4,237; 41.4%), followed by 0 to 4 (2,466; 24.1%) (Table 2). Median age was 22 years, ranging from 0 to 99.

A total of 9,848 (96.1%) patients had their rehydration plan recorded, of them, 4,239 (43.1%) were treated under plan A (no dehydration), 4,128 (41.9%) received plan B (moderate dehydration), and 1,481 (15.0%) were managed with plan C (severe dehydration). Females represented 47.4% of plan C, 50.6% of plan B and only 43.2% of plan A cases. Hospitalisation was required in 3,164 cases (32.1%), while 6,555 (66.6%) were managed as outpatients. There were 16 (0.2%) deaths, and 225 (2.3%) patients left against medical advice. Additionally, 114 (1.2%) were referred to another facility. Mostly, patients from Mafraq were referred to Mocha (80%), others were referred to non-MSF facilities for the treatment of comorbidities. The rest of the patients, 9,322 (94.7%), were discharged home (Table 2). Information on vaccination status was largely unavailable; only 4 patients (0.04% of all suspected cases) reported prior vaccination, of whom 3 presented a vaccination card. Similarly, malnutrition status was poorly documented: 237 patients (2.3%) were recorded as malnourished, while no information on screening was available for the rest. Moreover, symptom onset was only recorded in 4,244 (43.09%) patients.

Throughout the outbreak and in MSF-supported facilities, sample collection for bedside or laboratory testing was carried out in 707 patients (6.9%). Among these, 673 (95.2%) underwent rapid diagnostic testing (RDT), of which 444 (66.0%) were positive. Culture testing was performed in 128 cases, with 81 (63.3%) confirmed as positive. Remarkably, the results of 20 (15.6%) of those cultures were never marked in the patient’s file. A total of 94 patients underwent both RDT and culture, with 29 (30.8%) discordant results, being 16 (55.2%) positive RDT and negative culture testing and 13 (44.8%) positive culture and negative RDT.

Table 2 represents the patients with a recorded rehydration plan (*n* = 9,848). There was a higher proportion of laboratory samples taken from most severe cases, as 272 (18.4%) plan C cases were tested compared to 157 (3.7%) plan A and 302 (7.3%) plan B (Table 2).

**Table 2:** Characteristics of patients with different treatment plans in MSF-supported facilities in Aden, Ataq, Mafraq, and Mocha during the 2023–2024 outbreak in Yemen

Characteristic	Plan A		Plan B		Plan C		Total	
	*n*	%	*n*	%	*n*	%	*n*	%
Total	4,239	43.1	4,128	41.9	1,481	15.0	9,848	100
Sex								
Female	1,826	43.2	2,092	50.8	750	50.6	4,668	47.4
Male	2,401	56.8	2,029	49.2	731	49.4	5,161	52.6
Age group								
0 to 4	1,283	30.4	941	22.8	175	11.8	2,399	23.8
5 to 14	632	15.0	598	14.5	167	11.3	1,397	13.8
15 to 44	1,780	42.1	1,664	40.4	608	41.1	4,052	40.2
> 44	530	12.5	917	22.3	530	35.8	1,977	19.6
Laboratory results								
Sample collected	157	3.7	302	7.3	272	18.4	731	7.4
RDT performed ^a^	145	92.4	276	91.4	245	90.1	666	6.8
RDT positive ^b^	54	37.2	188	68.1	202	82.5	444	4.5
Culture performed ^a^	15	9.6	32	10.6	73	26.8	120	1.2
Culture positive ^b^	6	40.0	26	27.3	49	67.1	81	0.8
Centre of treatment								
Aden	1,458	34.4	2,710	65.6	1,190	80.3	5,358	54.4
Ataq	53	1.3	33	0.8	19	1.3	105	1.1
Mafraq	1,100	26.0	698	16.9	50	3.4	1,848	18.8
Mocha	1,628	38.3	687	16.6	222	15.0	2,537	25.7
Hospital stay								
Length in days (mean ± standard deviation)	1.3 ± 0.9		1.8 ± 1.2		2.2 ± 1.5			
Exit status								
Discharged	4,009	94.6	3,943	95.5	1,370	92.5	9,322	94.7
Died	3	0.1	2	0.0	11	0.7	16	0.2
Left against medical advice	147	3.5	64	1.6	14	0.9	225	2.3
Referred	51	1.2	29	0.7	34	2.3	114	1.2

Totals and percentages were calculated, eliminating the missing data from the denominator

^a^ Percentages calculated from samples collected

^b^ Percentages calculated from samples tested

### Geographical analysis

Patients treated across the four centres predominantly originated from distinct district clusters.

#### Aden

**Fig. 3:**
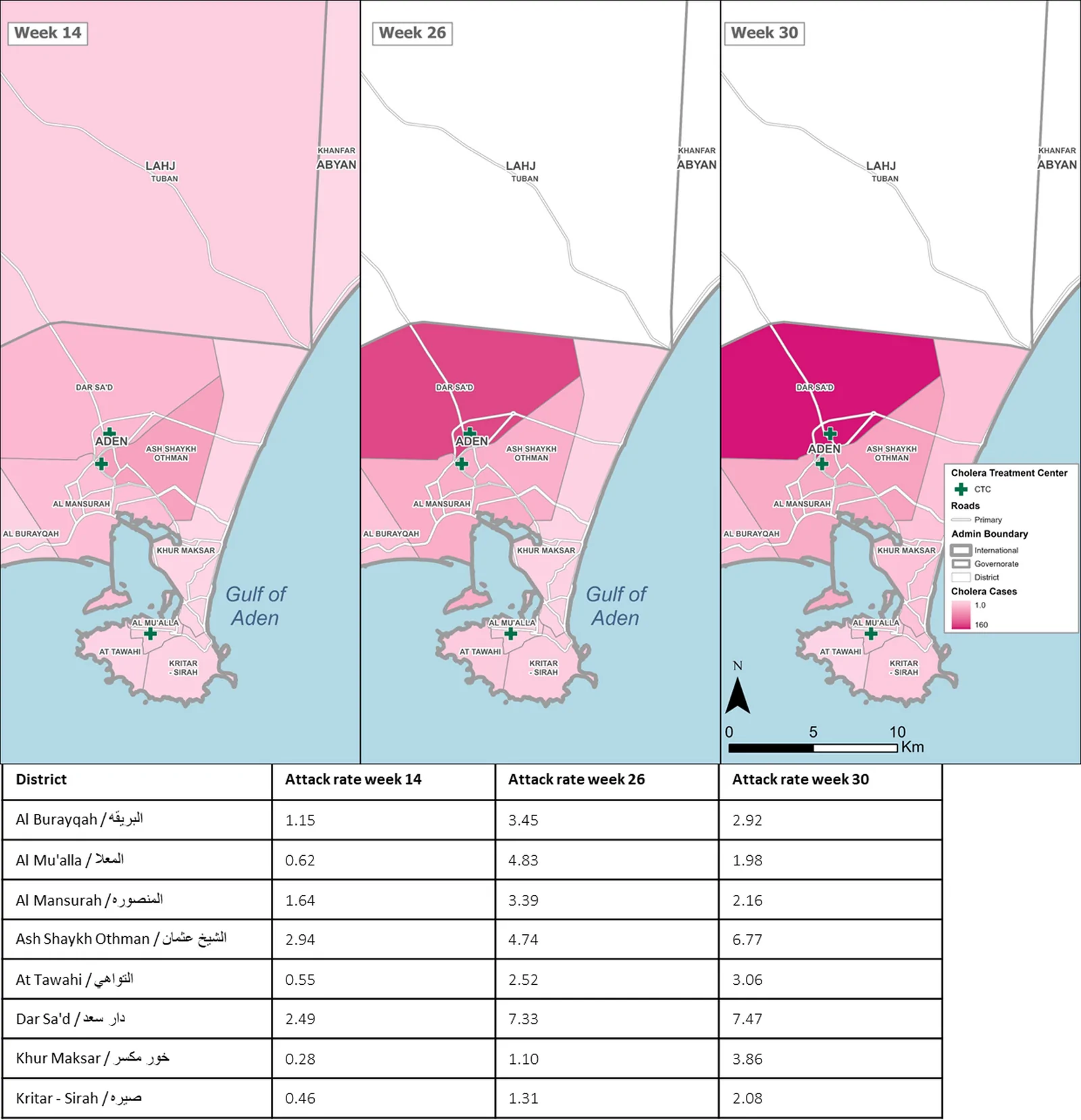
Weekly number of suspected cholera cases and attack rates treated at the Sadaqa Hospital (Aden) CTC, reported during 2024 epidemiological weeks of the outbreak

At the Sadaqa CTC, the majority of cases during the outbreak originated from urban districts within the Aden governorate itself, with Dar Sa’d (1,874; 35.0%), Ash Shaykh Othman (855; 16.0%), Al Mansurah (693; 12.9%), Al Burayqah (391; 7.3%), and Khur Maksar (176, 3.3%) consistently accounting for the highest patient volumes throughout the outbreak (Fig. 3). Notably, severe dehydration cases (plan C) were heavily concentrated in Dar Sa’d, a densely populated district located in the northern part of Aden governorate and serving as a key urban and residential area on the outskirts of central Aden.

**Fig. 4:**
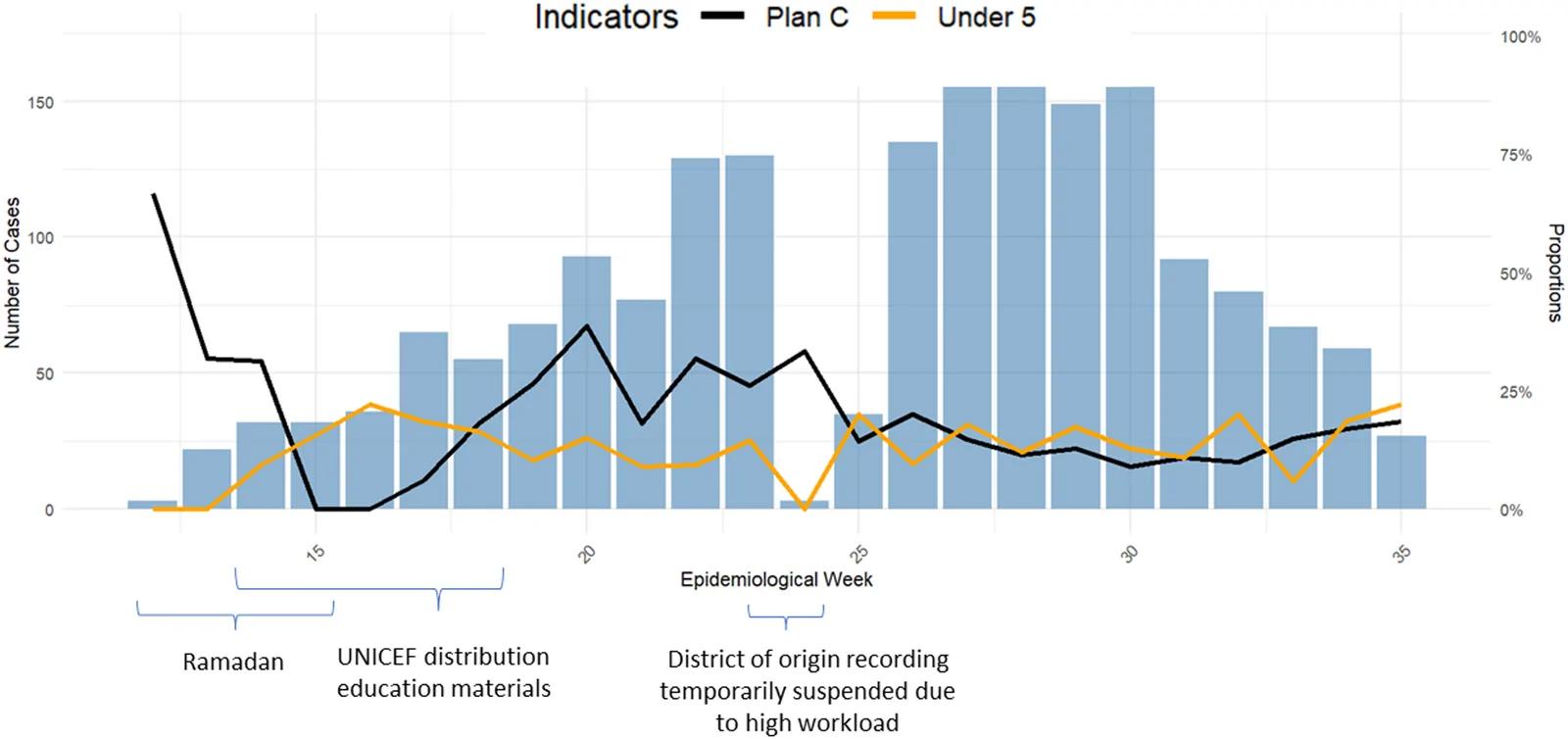
Epidemic Number of suspected cases, proportion of patients under 5 (orange), and proportion of patients on plan C (black), from Dar Sa’d District in 2024 epidemiological weeks 12–35 during the 2023–2024 cholera outbreak

The Aden Sadaqa CTC received 80% of all Plan C cases across all MSF-supported CTCs. Cases rose in week 14, coinciding with the end of Ramadan, and peaked between weeks 26 and 30 and then declined (Figs. 3 and 4).

The proportion of patients receiving plan C fluctuated, but generally showed higher proportions during early weeks. The proportion of children under 5 remained relatively stable, ranging from 15 to 25% (Fig. 4).

#### Mocha

**Fig. 5:**
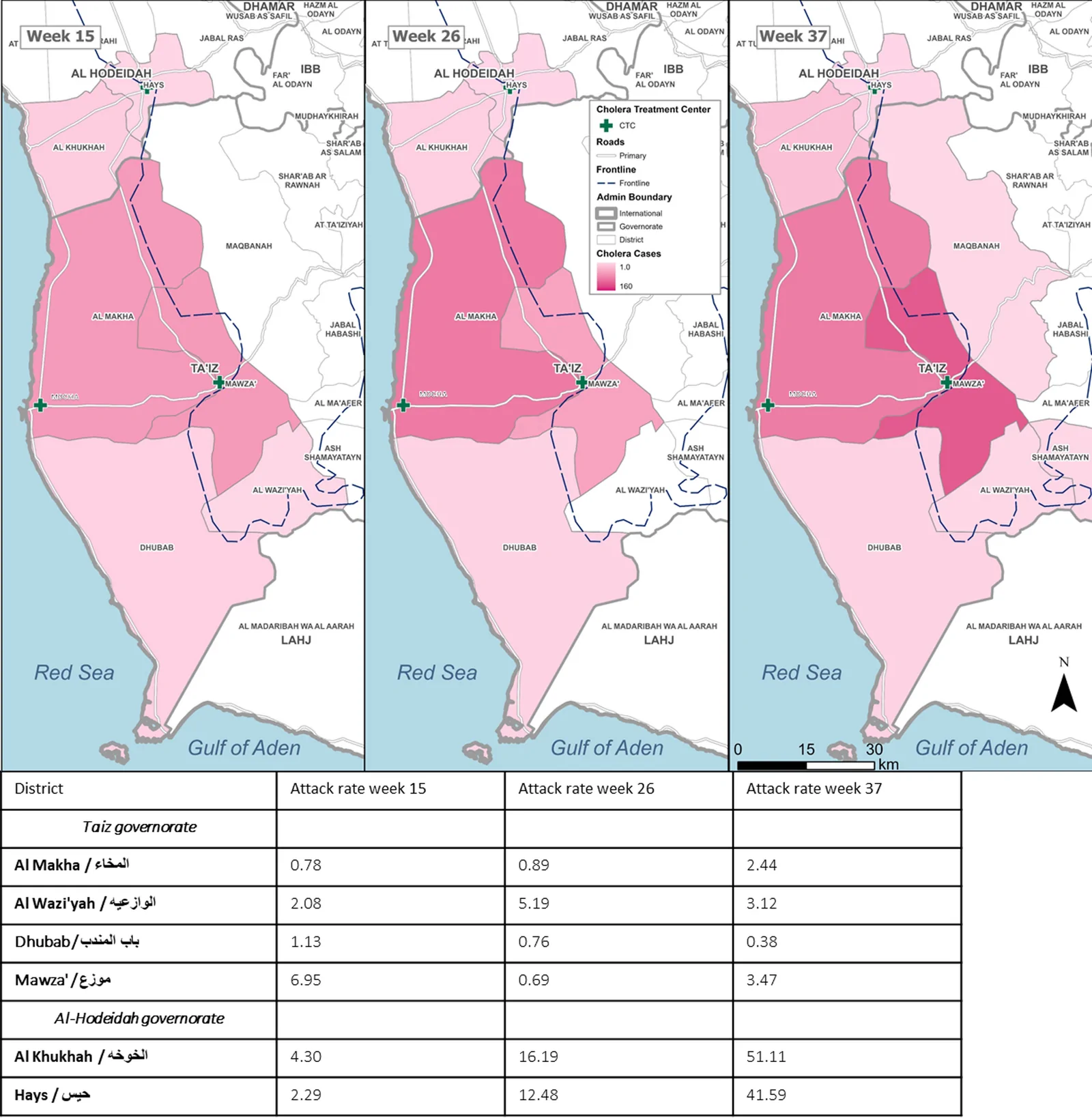
Weekly number of suspected cholera cases and attack rate in Mocha CTC, reported during 2024 epidemiological weeks of the outbreak

In the Mocha CTC, patients primarily came from the western districts of Ta’iz (2,537; 85.1%) and neighbouring Al Hodeidah governorate (377; 14.9%), mainly Al Khukhah district (332; 88.1%). In Ta’iz, the leading districts were Al Makha (1,721; 67.8%), Mawza’ (257; 10.1%), and Dhubab (149; 5.9%). Despite the active conflict, cases were received over the frontline (Fig. 5). The number of cases observed at Mocha Hospital extended over a longer period (Epidemiological weeks 15 to 39); although severe dehydration cases were documented, no particular district overwhelmingly contributed to plan C admissions.

**Fig. 6:**
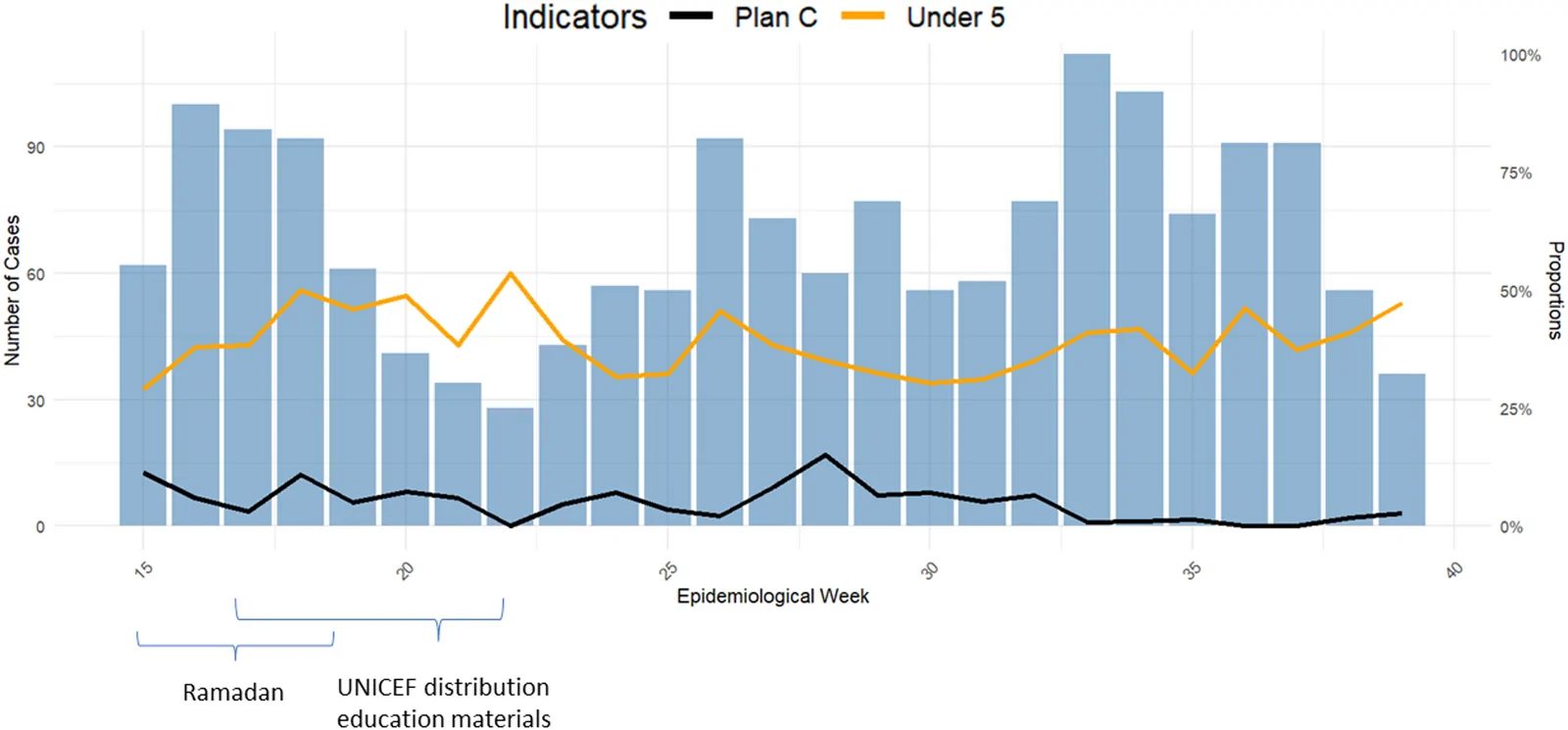
Epidemic Number of suspected cases, proportion of patients under 5 (orange), and proportion of patients on plan C (black), from Al Makha District in 2024 weeks 15–39 during the 2023–2024 cholera outbreak

The Mocha CTC experienced a rise in cases around week 15, followed by a first peak in week 26 and a second peak in week 32, with cases starting to significantly decline after week 37 (Fig. 5). Unlike a typical outbreak curve, Fig. 6 displays a prolonged, irregular transmission pattern rather than a single sharp peak. The proportion of patients receiving plan C fluctuated, but generally remained low. The proportion of patients under 5 was stable around 30%, which may be explained by the focus on the paediatric population of the Mafraq PHCC and Mocha hospital. It was not possible to conduct RDTs or cultures.

Mafraq PHCC CTU, a smaller MSF-supported site linked to Mocha’s referral network, served a mixed catchment area with most patients coming from Mawza (1,845; 99.8%), mainly from the subdistricts of Al Ahmul (1,662; 89.9%) and Mawza’ (172; 9.3%). Severe cases were sporadic, without clear geographical clustering.

#### Ataq

**Fig. 7:**
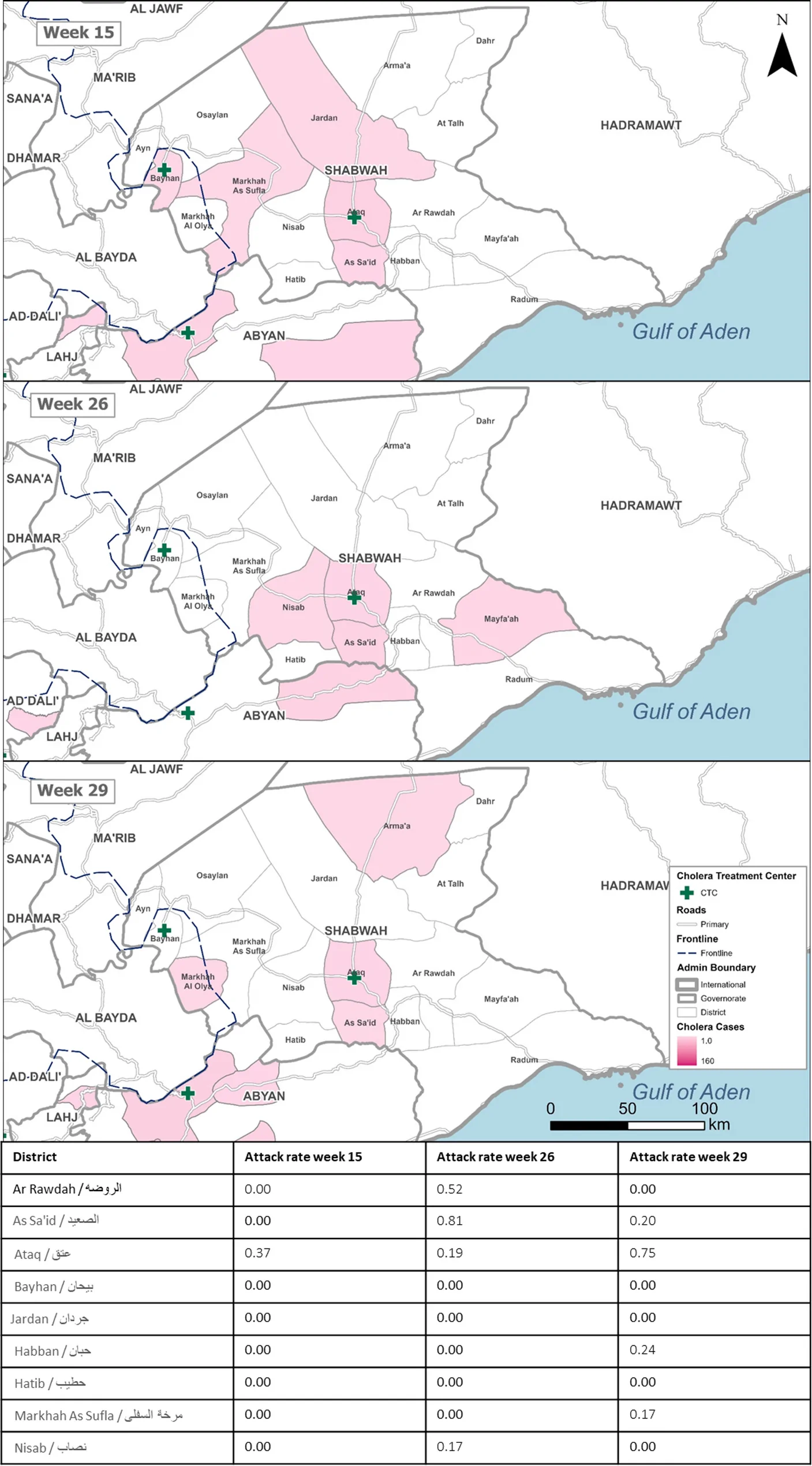
Weekly number of suspected cholera cases treated in the Ataq CTC, reported during 2024 epidemiological weeks of the outbreak

At Ataq CTC, the patient population mostly came from Ataq (56; 53.3%), As Sa’id (12; 11.4%), Al Mahfad (7; 6.7%) and Nisab (7; 6.7%) districts within the Shabwah governorate. These districts remained the principal sources of patients throughout the outbreak, although occasional admissions from neighbouring districts were recorded. Only a few cases were received from districts across the frontline (Fig. 7). Severe cases were evenly distributed among these districts.

**Fig. 8:**
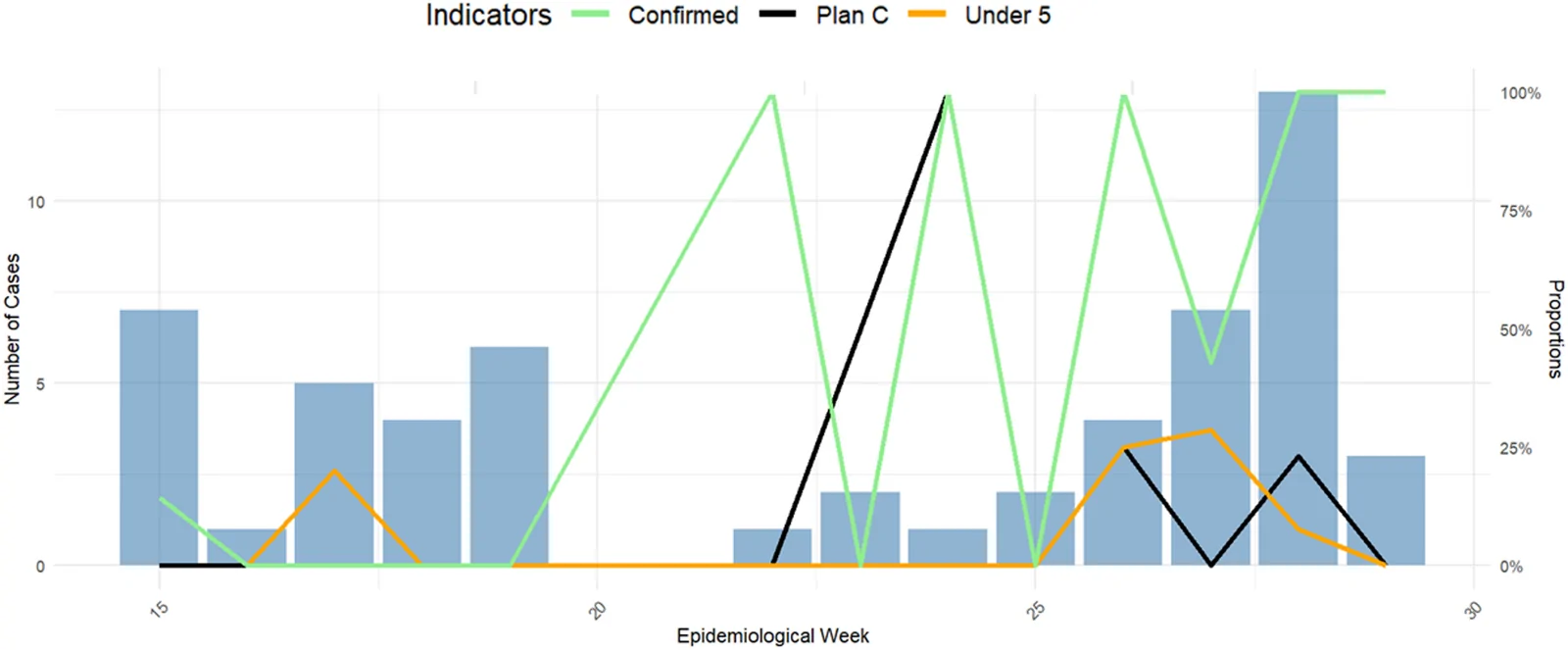
Epidemic Number of suspected cases, proportion of patients under 5 (orange), proportion of patients on plan C (black), and proportion of confirmed cases (green), from Ataq District in 2024 epidemiological weeks 15–29 during the 2023–2024 cholera outbreak

The Ataq CTC saw a rise in cases in 2023 and again in 2024, coinciding with the end of Ramadan. Data collection started in week 15. Cases started to rise in week 26 and gradually declined until week 29 (Figs. 2 and 7). The strong diagnostic capacity in Ataq enabled the testing of a high proportion of samples; the positivity rate fluctuated substantially from week to week (Fig. 8).

## Discussion

The 2023–2024 cholera outbreak in South Yemen presented a total of 40,000 suspected cases reported in South Yemen between April and October 2024, 25.6% of which were treated at MSF facilities, mostly in the Sadaqa Hospital CTC in Aden. The findings should be interpreted as a description of suspected cholera cases managed at MSF-supported facilities rather than a comprehensive epidemiological characterisation of cholera. The epidemic dynamics of suspected cases managed at MSF-supported facilities suggest a pattern consistent with sustained transmission with a rapid initial rise, a high plateau, and multiple waves, rather than a sharp peak, particularly in Aden and Mocha (Fig. 2). While cholera transmission is typically associated with contaminated water and food, our data do not allow direct assessment of transmission pathways or sources. Despite efforts to contain the outbreak, challenges remained in case management, laboratory testing, and targeted interventions. These challenges mirrored patterns observed during 2016–2018 and 2020 outbreaks, which affected over 2 million people [4, 8]. In 2016–2018, Camacho *et al*. reported 1,103,683 suspected cholera cases, with an AR of 3.69% and CFR of 0.22%. Moreover, they reported that Taiz governorate was among the highest-burden areas, with rapid transmission facilitated by conflict-related displacement and poor water, sanitation and hygiene (WASH) conditions [15]. The analysis of our 2024 data is consistent with these trends; however, our findings are limited to suspected cases managed at MSF facilities, and the interpretability of transmission dynamics is limited. Particularly in the districts of Taiz: Al Makha, Mawza’, and Al Khukhah, distinct catchment effects seem to be shaped by security frontlines and service availability. For example, our data showed that no cases were received in Mocha from Maqbanah district in 2024 (Fig. 5). This absence may reflect restricted population movement and referrals across the frontline due to active fighting. In Ataq primarily treated patients from the south of the governorate and a few from across the frontline, who appeared to avoid longer or unsafe referral routes (Fig. 7). In Aden, most cases originated from densely populated urban districts, mirroring the urban transmission focus observed by Camacho *et al*. [15]. These patterns may reflect treatment-seeking behaviour and access constraints shaped by security frontlines and service availability.

These patterns were further complicated by limited diagnostic preparedness. Our data showed a lack of a diagnostic strategy or capacity to implement it, with only 120 cultures performed throughout the outbreak period in the four sites and inconsistent RDT testing. Despite significant global RDT deployment in 2024 [28], testing was not evenly distributed across the epidemic in Yemen, due to demand unpredictability, limited stocks, and the lack of a proper diagnostic stewardship plan [29, 30]. Culture and RDT positivity rates were high early in the outbreak but could not be tracked consistently after week 21, because of irregular test availability, stock-outs, and the absence of a predefined testing strategy. This limited our ability to assess whether later suspected cases were true cholera or other diarrhoeal illnesses. Given these limitations, it is difficult to interpret whether changes over time reflect true shifts in cholera incidence or variations in case ascertainment. It is recommended to have a defined testing strategy at the emergency preparedness level and advocate for proper test capacity [30, 31]. Camacho *et al*. described a similar situation [15]. Despite six years having passed, the testing capacity remains very limited and unevenly distributed. Positivity rates in our data ranged from 27% to 83%, depending on method and severity group (Table 1). Despite these positivity rates fulfilling the expectations from other outbreaks (50–70%), the sampling criteria (or the lack of them) highlight bias (preferentially testing severe cases) and operational limitations. To monitor cholera incidence, systematic and representative sampling of suspected patients over time should be prioritised, rather than opportunistic or severity-based testing [31].

Several vaccination campaigns have taken place since 2018 [18–22]. However, due to a global vaccine shortage since 2022, only reactive campaigns with one dose were conducted. Due to coverage gaps and the shorter immunity of a one-dose schedule [32]. The full protective potential of the OCV was probably not reached during the study period. However, our dataset does not allow assessment of vaccination impact at the individual level.

Food insecurity and malnutrition are chronic issues in Yemen and a well-recognised risk factor for exacerbating the severity of diarrhoeal illnesses [12, 33]. It is estimated that 2.7 million children in Yemen are acutely malnourished, and 49% of children under 5 suffer from stunting or chronic malnutrition [17]. However, our data does not allow for assessments of malnutrition’s role in cholera. Furthermore, in this context, it is possible that some clinically suspected cholera cases in our dataset represented other causes of acute diarrhoea, which malnourished children are especially vulnerable to. Misdiagnosis is possible given overlapping clinical presentations and limited laboratory confirmation. This is further suspected due to the high proportion of plan A patients, particularly towards the end of the outbreak. However, our data on malnutrition did not allow us to draw any conclusions. Systematic nutritional and vaccination screening should be strengthened in future outbreaks. This would require staff training and improvement of OCV understanding and reporting (ideally by stamped cards).

While opening new CTCs/CTUs [19] and ORP (198) to respond to this outbreak represented a significant scaling-up, care provision was still concentrated in major facilities like Sadaqa CTC. Limited decentralisation of care and weak mortality surveillance at the community level raise the possibility that deaths occurring outside facilities were not captured [31, 34]. The 0.52% CFR reported in South Yemen, could underestimate deaths outside facilities, particularly in rural catchments where delayed care‑seeking and access barriers are common contributors to mortality. In future outbreaks, symptom onset dates should be systematically recorded to better assess whether patients from more remote areas arrive later and sicker.

The increase in reported cases coincided with Ramadan; however, this study cannot determine whether Ramadan-related behaviours contributed to transmission or healthcare-seeking patterns. The onset of the epidemic in Aden aligned temporally with Ramadan (March-April 2024), a period associated with large gatherings, communal meals, and potentially reduced health-seeking behaviour during fasting hours. Although its correlation to cholera remains to be further studied [35]. Such delays in accessing care could partly explain the early increase in the proportion of severe cases observed, as cholera can progress rapidly from mild symptoms to life-threatening dehydration [36]. However, our dataset does not include sufficiently complete symptom onset data to assess its contribution to severity patterns. UNICEF educational materials and health-promotion activities occurred during the outbreak period [23], but the present data cannot determine whether these activities affected healthcare-seeking behaviour or contributed to the subsequent decline in reported cases.

The 0.52% CFR during the 2024 cholera outbreak in South Yemen aligns with previous outbreaks in Yemen, where CFRs typically ranged between 0.2% and 0.6% [15]. In MSF-supported facilities specifically, the CFR was even lower at 0.2%. In 2016–2018, severe dehydration rates varied by setting but were generally under 20%. In our 2024 dataset, severe dehydration (plan C) accounted for 15% overall, but with notable clustering—Aden’s Sadaqa CTC managed 80.3% of all plan C cases, highlighting its role as a referral hub for the most critical patients and indicating that the distribution of severe cases across sites is strongly influenced by referral pathways rather than reflecting underlying epidemiological differences. We believe that this concentration primarily reflects catchment configuration and referral pathways, introducing referral bias that limits comparability between sites. Moreover, the high proportion of laboratory sampling from plan C patients in Mocha (18.4% vs. 3.7–7.3% for Plans A/B) suggested a severity-linked sampling bias similar to the targeted confirmatory testing practices previously described [15]. Compared with the Camacho 2016–2018 outbreak (1.1 million cases, AR 3.69%, CFR 0.22%), Yemen’s 2024 outbreak showed a lower AR (0.78%) but a slightly higher CFR at the national level (0.34%). Whether this reflected improved management, persistent access gaps or an under-representation of milder cases is uncertain, since untreated patients outside the system remain invisible.

Despite the involvement of numerous UN agencies, international and local NGOs and the efforts of the MoH, cholera outbreaks remained challenging in Yemen. Outbreak management still relied primarily on syndromic reporting due to the lack of an active laboratory-based surveillance. In the 2023–2024 outbreak, South Yemen focused on improving WASH and vaccination coverage, although both were limited mainly due to the ongoing conflict and the global shortage of oral cholera vaccines (OCV) [9, 37]. The WASH and health promotion campaigns carried out included the distribution of more than 70,000 hygiene kits and awareness-raising campaigns that reached more than 250,000 people [38, 39]. Yet, these efforts focused on harm reduction, rather than prevention, and so did not improve the water and sanitation infrastructure to a point that could reduce the frequency of outbreaks, letting cholera remain endemic in Yemen. To strengthen future outbreak management, it is recommended to prioritise continuous capacity-building of healthcare workers through regular training on case detection, management, and infection prevention and control. Case definitions and testing strategies should be adapted during outbreaks to reflect changing epidemiology, to distinguish cholera from other acute watery diarrhoea and design targeted public health responses. Preparedness for future outbreaks is critical. Maintaining strategic stocks of RDTs, medication, and vaccines amidst global shortages should be prioritised. Moreover, water quality monitoring and awareness-raising campaigns should be sustained year-round rather than limited to emergency phases. In summary, outbreak preparedness must shift from reactive to anticipatory approaches, with a focus on preventive interventions and clear guidance on allocation and use, ideally through global stockpiles rather than fragmented national reserves. Without durable improvements to water and sanitation infrastructure, outbreaks will continue, especially under conditions of conflict and displacement.

### Limitations

This is a retrospective description of suspected cholera cases managed at MSF-supported facilities, and not a comprehensive epidemiological characterisation of cholera. Due to the retrospective nature of this report, it was limited by the data quality collected during the outbreak, with no possibility of improving the completeness of the database. Variables not explored due to missing values were vaccination status, symptoms and malnutrition, which prevented our reflection on their impact on severity. Moreover, information on complementary therapy: doxycycline or zinc supplementation was inconsistent. In addition, our sample might have been prone to selection bias as the patients who presented at MSF CTC/CTUs may have significantly differed from those who did not want or could not reach the facilities. Furthermore, patient triage and management could vary slightly across facilities, limiting the comparability of the data. This does not give the full picture of the national scale of the outbreak, but rather a limited representation of what was seen in the MSF-supported facilities.

## Conclusion

The 2023–2024 cholera outbreak in South Yemen underscored the persistent challenges of cholera control in conflict settings. Despite effective case management and a low case fatality rate, cases were reported over several months. Outbreak preparedness should be strengthened, particularly through a decentralised network of CTCs, CTUs, and ORPs, a rational diagnostic strategy, enhanced surveillance, and systematic data collection. Investment in vaccination and water and sanitation infrastructure also remains critical to reducing the frequency and impact of future outbreaks.

## Data Availability

Data are available on request in accordance with the MSF Data Sharing Policy. Requests can be directed to [data.sharing@msf.org](mailto: data.sharing@msf.org) .

## Abbreviations

CEmONC: Comprehensive emergency obstetric and newborn care
CFR: Case fatality rate
CI: Confidence intervals
CTC: Cholera treatment centres
CTU: Cholera treatment units
eDEWS: Yemen’s electronic Disease Early Warning System
IOM: International Organization for Migration
LAMA: Left against medical advice
LuxOR: Luxembourg Operational Research
MEMU: Middle East Medical Unit
MoH: Ministry of Health
MSF: Médecins Sans Frontières, Doctors Without Borders
OCV: Oral cholera vaccine
ORP: Oral rehydration points
ORS: Oral rehydration solution
RDT: Rapid diagnostic test
TCBS: Thiosulfate citrate bile salts sucrose agar
WHO: World Health Organization
